# Pollution and respiratory disease: can diet or supplements help? A review

**DOI:** 10.1186/s12931-018-0785-0

**Published:** 2018-05-02

**Authors:** T. Whyand, J. R. Hurst, M. Beckles, M. E. Caplin

**Affiliations:** 1 Centre for Gastroenterology, Royal Free Hospital, London, NW3 2QG UK; 2 UCL Respiratory, University College London, London, UK; 3 Department of Medicine, Royal Free Hospital, London, UK

**Keywords:** Pollution, Diet, Lungs, Supplements, Asthma, COPD, Smoke, Particulates, Vitamins, Omega-3, Curcumin

## Abstract

Pollution is known to cause and exacerbate a number of chronic respiratory diseases. The World Health Organisation has placed air pollution as the world’s largest environmental health risk factor. There has been recent publicity about the role for diet and anti-oxidants in mitigating the effects of pollution, and this review assesses the evidence for alterations in diet, including vitamin supplementation in abrogating the effects of pollution on asthma and other chronic respiratory diseases. We found evidence to suggest that carotenoids, vitamin D and vitamin E help protect against pollution damage which can trigger asthma, COPD and lung cancer initiation. Vitamin C, curcumin, choline and omega-3 fatty acids may also play a role. The Mediterranean diet appears to be of benefit in patients with airways disease and there appears to be a beneficial effect in smokers however there is no direct evidence regarding protecting against air pollution. More studies investigating the effects of nutrition on rapidly rising air pollution are urgently required. However it is very difficult to design such studies due to the confounding factors of diet, obesity, co-morbid illness, medication and environmental exposure.

## Background

The World Health Organisation (WHO) released a report in 2014 indicating that 3.7 million premature deaths globally were attributable to ambient air pollution [1]. Their data more than doubled previous estimates and placed air pollution as the world’s largest environmental health risk factor [1]. The majority of outdoor pollutants come from anthropogenic sources such as vehicle emissions, fossil fuel combustion, forest fires and industrial processes including factory outputs [2]. The WHO showed that in urban areas which monitor air pollution levels, greater than 80% of people are exposed to levels of pollution which exceed WHO limits [1]. Primary pollutants can be divided into two groups: particulate matter (PM) and gases (CO2, CO, NO2, NO, NOx, SO2) [2]. Secondary pollutants such as ozone are formed from photochemical reactions between the primary pollutants, heat and UV radiation. Other environmental air pollutants of major public concern include polycyclic aromatic hydrocarbons (PAHs) and aryl hydrocarbon receptors (AhR).

There has been increasing research on the effects of ambient pollution on health. Pollution causes damage when it is in contact with the airways and skin. For example, some pollutants can accumulate in the blood and be distributed in digestive organs, purely through inhalation [3]. Pollutants also act on the exterior of the body and have been linked to the progression of inflammatory skin diseases [2, 4–9]. Many studies have demonstrated the effects of exposure to environmental pollutants via skin, inhalation or ingestion on morbidity and mortality [9, 10].

The lungs rely on filtered air through the nose (with cilia and mucus attempting to filter/trap unwanted particles) or unfiltered air via the mouth. Polluted air contributes to chronic obstructive pulmonary disease (COPD) prevalence and symptom onset [11]. The idea that air pollution can cause exacerbations of pre-existing asthma is supported by an evidence base that has been accumulating for several decades [12–15], however it has more recently been suggested that air pollution might cause new-onset asthma as well [16–26].

In October 2013, a Working Group of invited experts from 11 countries met at the International Agency for Research on Cancer (IARC) in Lyon, France, to evaluate the carcinogenicity of outdoor air pollution. The Group unanimously classified outdoor air pollution and PM from outdoor air pollution as carcinogenic to humans (IARC Group 1) based on sufficient evidence of carcinogenicity in humans and experimental animals and strong mechanistic evidence [27].

In addition to outdoor air pollution, indoor smoke is a serious health risk for some 3 billion people who cook and heat their homes with biomass fuels and coal. Some 4.3 million premature deaths were attributable to household air pollution in 2012. Almost all of this burden is in low-middle-income countries [28]. When indoor and ambient air pollution are combined, WHO estimates that in 2012, some 14% of deaths were due to COPD or acute lower respiratory infections, and 14% of deaths were due to lung cancer [29].

There has been recent publicity on the role of diet helping to combat the effects of pollution and in this review we assess the role for diet in preventing the effects of pollution on asthma and other respiratory diseases.

## Method

In 2017 to March 2018, a search of ‘air pollution and diet’, ‘pollution and diet’, ‘pollution and antioxidants’, ‘pollution and fats’ ‘pollution diet and lung disease’ ‘pollution and respiratory disease’, ‘pollution and lung cancer’, ‘metabolism and air pollution’, ‘obesity and air pollution’, ‘Mediterranean diet and air pollution’, ‘Western diet and air pollution’ was conducted using PubMed. A search of original research and review papers from the past twenty years, dating back to 1997 resulted in 109 relevant papers of mainly original research being reviewed.

### Overview of pollutants

#### Phthalates

It is known that phthalates are widespread contaminants in both indoor and outdoor environments with the plastic industry being a major contributor. They are mainly added to plastics to increase their flexibility, transparency, durability, and longevity. They are used primarily to soften polyvinyl chloride (PVC). Studies suggest that diethylhexyl phthalate (DEHP), a high molecular species used in plastic wrapping of foods, is a major source of exposure for humans as a result of contamination from the packaging, an effect made greater with microwave heating. The toxicants can be delivered into the body via inhalation, dietary intake, and skin absorption inducing an inflammatory response [29]. Most experimental studies address the adjuvant effects of phthalates in immune responses [30] and they may contribute to airway remodelling [31] and affect respiratory health [32–36].

#### Particulate matter

PM is a complex mixture of particles found in the air, including dust, dirt, soot, smoke, and liquid droplets particles suspended in air and are produced by a variety of natural and anthropogenic activities [10]. Major sources of PM include open fires, industrial facilities, power plants and vehicle exhausts [10]. PM can be divided into three types depending on size; ultrafine particles (UFP), fine particles (PM2.5) and coarse particles (PM10). Due to the increase in urbanisation and industrial processes PM are widely implicated in contributing to ambient pollution worldwide and are associated with increased morbidity and mortality [10]. PM can penetrate the alveolar regions of the lung, pass through the cell membrane, reach the blood and can accumulate in other human organs [3]. Additionally, epidemiological investigations into contamination, especially ambient air pollution, indicated that the PM is not only correlative with the exacerbation of cardiovascular diseases and respiratory systemic inflammation, but also the progression of inflammatory skin diseases [2] such as atopic dermatitis (AD) [4–6], acne, psoriasis, and allergic reactions [7–9]. The metabolic effects of PM_2.5_ are also evident with significant increases in carcinoembryonic antigen and fasting blood glucose, and significant decreases in HDL cholesterol in Chinese policemen who work at least 1 hour a day outside for 1 year [37]. Additionally, PM_2.5_ inhalation reduces ATP production by disrupting the aerobic tricarboxylic acid cycle and oxidative phosphorylation, thereby causing the hypophosphorylation of tau in the cortices of middle-aged mice. Excessive reactive oxygen species generation was involved in the impairment, but interestingly, these alterations were partially reversed after exposure to PM_2.5_ had ended [38].

PM can induce oxidative stress and inflammation on respiratory organ tissue [39–42] exacerbating asthma [43, 44] and COPD [45–47]. Dust particles alone may mediate airway inflammation, the progression of asthmatic diseases [48] and pneumonia [49, 50]. PM from different fuels and combustion phases have appreciable differences in lung toxic and mutagenic potency, and on a mass basis, flaming samples are more active, whereas smouldering samples have greater effect when emission factors are taken into account [51]. In another study, coarse PM from roadside air elicited a genotoxic response in the normal alveolar cell lines [52]. There are various population studies, and many from China, for example; an average of 23.1% lung cancer burden was attributable to PM2.5 pollution in Guangzhou during 2013 [53]. In the US Adventist Health and Smog Study-2 (AHSMOG-2) study increased risk estimates of lung cancer were observed for each 10-μg/m^3^ increment in ambient PM_2.5_ concentration. The estimate was higher among those with longer residence at enrolment address and those who spent > 1 hr/day outdoors [54].

#### Polycyclic aromatic hydrocarbons

PAHs are generated during the incomplete combustion of organic matter and can form complex mixtures with airborne particulate matter or gases. The majority of outdoor PAHs are derived from coal tar, diesel exhausts and cigarette smoke. PAHs may be metabolically activated to generate reactive oxygen species (ROS) that can react to form bulky DNA adducts or strand breaks on cellular DNA [55]. PAH exposure has been linked to adverse respiratory health outcomes in children, including bronchitis [56, 57] and reductions in the forced expiratory volume, (FEV_1_) [58–62]. Among adults in occupational settings, elevated PAH exposures have been found to be associated with declines in FEV_1_/forced vital capacity (FVC) [63]. In an in vitro animal cell model, low molecular weight PAHs and benzo[a]pyrene (which are found together in cigarette smoke) elicited increased carcinogenic potential [64].

#### Ozone

Ground level ozone (O3) generation is a major component of smog and is formed as a result of a photochemical reaction between O2 and pollutants such as hydrocarbons and nitrous oxides, which is facilitated by sunlight. Excessive ozone in the air can have a marked effect on human health. It can cause breathing problems, trigger asthma, reduce lung function and cause lung diseases such as COPD [28]. Short-term exposure to ozone is associated with respiratory morbidity and mortality [65–68]. Long-term exposure has been linked to premature respiratory mortality in adults [69] and to increased risk of death in susceptible populations with chronic cardiopulmonary diseases and diabetes [70]. Less evidence is available for an association between ozone and lung cancer, with one Canadian study finding an odds ratio for lung cancer incidence of 1.09 (0.85–1.39) with a 10-U increase in ozone [71] and another Canadian study found ozone was a non-significant contributor to lung cancer mortality [72].

#### Nitrogen dioxide

Ambient pollution is also characterised by increased levels of nitrogen dioxide (NO_2_) and it is regarded as a strong marker for air pollution primarily generated from combustion including motor vehicles, biomass burning, airports and industry [73, 74]. A recent meta-analysis of 13 studies from across North America, Europe and Asia has shown a modest increase in the risk of respiratory mortality with increasing chronic exposure to NO_2_ [74, 75]. Exposure to ambient NO_2_ increases systemic inflammation in COPD patients, especially in former smokers [76]. Each 10 μg/m^3^ increase in nitrogen dioxide corresponded to an increased risk for diagnosed asthma in 6–13-year-old Chinese children [77].

#### Persistent organic pollutants

Persistent organic pollutants (POPs) comprise a large variety of substances such as Polychlorinated biphenyls (PCBs) and Polybrominateddiphenyl ethers (PBDEs). PCBs are a persistent public health threat in indoor environments, because they were purposefully added to household sealants, paint plasticizers, wood finishes, flame retardants, light ballasts, and electrical capacitors in appliances [78, 79]. Inadvertent production of PCBs is an additional, emerging concern. PCBs are present in modern pigments used in household paint and many consumer products [80, 81]. PCBs are also present in outdoor environments due to contributions from contemporary urban sources, [82, 83] and, to a lesser amount, volatilisation from soil and water bodies [84–86].

PCBs are highly lipophilic; bioaccumulate in fats, lipids, and waxes; bioconcentrate in food chains; and are semi-volatile. In view of high PCB concentrations in some animals, studies of dietary PCB exposure have historically taken precedence over dermal and inhalation exposure. However, airborne emissions from newly produced PCBs may lead to inhalation exposure at levels comparable to, and sometimes higher than, dietary exposure [87–89]. There is also rising evidence that some PCBs are mutagenic and tumour promoting [90]. For example 2 cohorts of highly exposed populations in Asia developed high rates all cancers, lung cancer and liver disease (men) and liver cancer (women) [91]. This may be because PCBs appear to contribute to oxidative damage in the body [92–97].

#### Mixed pollutants

Air often contains a mixture of pollutants. In Chinese children, mixed pollutants (PM10, sulphur dioxide, nitrogen dioxides, and O3) have been linked to increased blood pressure, and obesity seemed to amplify these changes [98]. Another team however feel that the inhalation of traffic related air pollution causes obesity, by reducing children’s physical activity and through inflammatory pathways [99]. There is also a relationship to inflammation initiating metabolic processes involved in diabetes development [100, 101].

Environmental tobacco smoke and chemical emissions from new furniture are risk factors for asthma [102] and for wheeze and daytime breathlessness [103]. Males without allergic predisposition and females with allergic predisposition had increased susceptibility to the adverse impact of air pollution on asthma [16]. Several other studies have also indicated that the airways of males and females respond differently to exposure to air pollutants [104–107]. This is plausible as there are differences between male and female airways from early in foetal lung development and throughout life [108, 109], for example female lungs mature earlier with regard to surfactant production. Throughout life, women have smaller lungs than men, but their lung architecture is more advantageous with a greater airway diameter in relation to the volume of the lung parenchyma. Thus, in childhood, airway hyper-responsiveness and asthma is more common among boys than girls. Among the children without an allergic predisposition, the stronger association between ambient air pollutants exposure and respiratory symptoms and diseases in males, could be related to males having less mature lungs and relatively narrower airways during childhood. These factors are believed to contribute to the generally higher rates of pulmonary morbidity in boys than in girls, and could possibly also explain a higher susceptibility for damage by exposure to air pollutants during this age window. Increased ambient ozone, NO_2_, PM2·5, and sulphur dioxide (SO_2_) levels were associated with increased hospital admission for asthma [110–114].

Many developing countries or regions (eg, rural China, India, South America and Sub-Saharan Africa) still use open fires for cooking or heating and despite mixed findings for biomass smoke exposure and asthma risk, coal combustion for heating and cooking conferred higher risks of childhood asthma in China [115]. In multi-pollutant models, black carbon conferred greater asthma admission risks for children, [116]. Moreover, the adverse effects and resulting diseases such as COPD from smoke pollution could be long lasting as the lungs are still developing into a person’s early twenties. Another study demonstrated that each 15μg/m^3^ and 50 μg/m^3^ increase in NO_2_ and SO_2_ levels respectively was associated with an increased risk of asthma, whereas combined exposure to high levels of NO_2_ and SO_2_ further increased asthma risk in the first year of life [117]. A report in the USA has shown that early-life exposure to traffic-related air pollution correlated with FEV_1_/FVC [118]. Groups of pollutants have already been discussed in terms of their carcinogenic abilities, and it is clear that causes of lung cancer are changing. A study of 495 lung cancer patients in the Indian sub-continent found that incidence was higher among non-smokers [119]. This shows that the focus within lung cancer prevention needs to move from anti-tobacco public health messaging, to wider national and international policies to clean up air we are forced to breath.

### Nutrient protection

Oxidative stress plays an important role in the development of age-related diseases [120].Evidence increasingly suggests that poor diet, including clinical malnutrition may increase the risk for oxidative stress and chronic diseases [92–97, 120]. Nutrition is known to play a significant role in the prevention and management of these same chronic diseases and has been shown to modulate the toxicity of PCBs [93–97]. One study has suggested that there may be increased susceptibility to NO_2_ when someone is in a fasting state [121] but it is not known if it is the same for other pollutants.

Oxidative stress, resulting from an imbalance between reactive oxidant species and antioxidants, can lead to tissue damage, airway inflammation with increased asthma severity and abnormal immune responses [122–124]. Serum concentrations of antioxidants have been positively associated with FEV_1_ in people with and without asthma [125, 126]. Vitamin, mineral and botanical compounds, with and without antioxidant properties will be discussed.

#### Vitamin A and carotenoids

Vitamin A contributes to key biological processes including growth, vision, epithelial differentiation, reproduction, and immune responses [127]. The two dietary sources of this vitamin are pre-formed vitamin A (retinol) and pro-vitamin A (carotenoids) [128]. Dietary intake of retinol comes from animal sources (eg, whole milk, liver, and eggs) and fortified foods. Orange and yellow fruits and vegetables (eg, carrots) are the main dietary sources of carotenoids, including α-carotene, β-carotene, lycopene and β-cryptoxanthins and they are known antioxidants [129]. Oxidative stress might exacerbate asthma by increasing the release of pro-inflammatory cytokines, which may lead to increased airway inflammation, and airway responsiveness [122, 123, 130]. Vitamin A might improve prevention or treatment of asthma by downregulation of oxidative stress [131], or via direct effects on the immune system [132] for example downregulation of T-helper (Th)2 (pro-allergic) immune responses [133, 134]. However, vitamin A also enhances protective Th2 immune responses (eg, interleukin (IL) 4 expression) [135]. Lycopene, (a carotenoid) supplementation was shown to reduce allergic airway inflammation, [136]. Self-reported dietary intake of vitamin A or its components (retinol and carotenoids) was inversely associated with asthma and asthma severity. However, dietary intake of vitamin A was not significantly associated with wheeze or airway responsiveness [137].

No primary randomised controlled trial (RCT) of vitamin A for the prevention or treatment of asthma has yet been performed. There are a number of studies of vitamin A supplementation, but such studies were difficult to control for dietary intake, and gave variable results [138–140]. However, higher intake of tomatoes, carrots, and leafy vegetables (which are rich in carotenoids such as α-carotene, β-carotene, lutein, and zeaxanthin) has been associated with lower prevalence of asthma in women [141]. Of the pro-vitamin A carotenoids, β-carotene has been most intensely studied because of its protective properties against the effects of free radicals and oxidative damage. A meta-analysis of five observational studies showed that high dietary intake of β-carotene was not significantly associated with asthma or FEV_1_ [142]. However, this meta-analysis was limited by sample size and residual confounding. Results of two RCTs showed that modification of dietary intake of carotenoids through a diet rich in fruits and vegetables [143] or lycopene-rich tomato extract and tomato juice [144] improved indicators of asthma control, including lung function measures and time to disease exacerbations, in adults with asthma. Larger RCTs (including both children and adults) with longer follow-up are needed to further validate these findings.

According to World Cancer Research Fund/American Institute for Cancer Research (WCRF/AICR) Second Expert Report from 2007, foods rich in carotenoids may protect against lung cancer (strength graded as ‘probable’) [145]. However, in contrast, two large randomized double-blind placebo-controlled trials, the alpha-tocopherol-*β*-carotene (ATBC) and the *β*-carotene and Retinol Efficacy Trial (CARET) showed an increased risk of lung cancer among high-risk people supplemented with high doses of *β*-carotene and/or *α*-tocopherol [146–149]. The latest meta-analysis showed higher blood concentrations of total carotenoids, *α*-carotene, *β*-carotene, lycopene, and retinol were inversely associated with lung cancer risk [150]. However, because of the lack of data in people who never smoked, further large scale studies stratified by smoking status are needed to rule out residual confounding by smoking.

#### Vitamin C and E

Observational studies have reported that low vitamin C and vitamin E intakes are associated with a higher prevalence of asthma. [151]. In the 2004 Cochrane database review, supplementation of vitamin C was not believed to have clinical benefits in people with asthma [152] although this, and later reviews were withdrawn as there were serious data errors.

Exposure to O_3_ results in dose dependent depletion of antioxidants vitamin C and E in the skin [153]. Antioxidant supplementation with vitamin C and E above the minimum dietary requirement led to attenuated nasal inflammation and partially restored antioxidant levels in asthmatic patients exposed to high levels of O_3_ [154]. A meta-analysis of 24 observational studies in children and adults found lower dietary intake (but not serum level) of vitamin E was also significantly associated with increased asthma severity [155]. A review of 15 observational studies (including 3 birth cohorts), suggested that the evidence linking low vitamin E to asthma development was methodologically weak but sufficiently supportive of a potential effect warranting follow-up in clinical trials [156].

A RCT found no effect of vitamin E supplementation (500 mg/day) for six weeks on airway responsiveness in 72 British adults with asthma [157]. A second RCT found no effect of an antioxidant supplement (containing β-carotene, vitamin C and vitamin E) on plasma F2-isoprostanes, exhaled nitric oxide (NO) or peripheral blood mononuclear cell (PBMC) immune responses in 54 allergic adults [158]. In contrast to these negative results, four RCTs reported that vitamin E-containing antioxidants reduce O_3_-induced bronchoconstriction in subjects with [159, 160] and without [161, 162] asthma, suggesting potential protective effects of vitamin E against the detrimental effects of O_3_. A study in children looked at total antioxidant intake and asthma rates, and there appeared to be a link between higher antioxidant intake (total antioxidant capacity) and diminished sensitivity to inhaled allergens. Children in areas of low traffic pollution displayed a stronger association between sensitivity to allergens and antioxidant capacity [163]. In people with exercise induced asthma, vitamin C and Vitamin E supplement aided recovery by improving flow rates [164]. A London based bidirectional case cross-over study looked at whether individual plasma antioxidant concentrations (uric acid and vitamins C, A, and E) and 10 antioxidant genes could modify the response to PM with respect to hospital admissions for COPD or asthma. Two hundred and thirty four admissions were recorded and the level of PM10 was noted 14 days before and after each event. Combined admission rates were related to a 10 μg/m increase in PM10. Serum vitamin C modified the effect of PM10 on asthma/COPD exacerbations. A similar (although weaker) influence was observed for low levels of uric acid and vitamin E, whereas vitamin A showed no effect modification [165].

In terms of cancer, one team demonstrated that vitamin E intake is a protective factor against lung cancer [166] whereas other studies suggested vitamin E intake has no effect on lung cancer [167–169]. Moreover, a different study showed that vitamin E intake increased the risk of lung cancer [170]. In a meta-analysis of 9 prospective cohort studies of vitamin E and lung cancer from 1955-2015 found that for every 2 mg/d increase in dietary vitamin E intake, the risk of lung cancer decreased by 5% [171]. A later single prospective cohort study in Japan found that vitamin C and E intake had no effect on lung cancer risk [172].

#### Vitamin D

Vitamin D is key to the metabolism of calcium and phosphorus. In adults with Asthma, normal vitamin D levels correlated with improved asthma control and therefore supplementation may play a role in uncontrolled asthmatics with vitamin D deficiency [173].

Irrespective of the threshold used, in a US cohort, reduced serum or plasma vitamin D concentrations are commonly detected in children and adults particularly in subgroups at high risk for asthma or asthma morbidity [174, 175].

Three population-based studies (two cross-sectional [174, 176] and one longitudinal [177] showed an association between reduced serum vitamin D concentrations and severe disease exacerbations or core measures (eg, hospitalisations) of severe exacerbations in Costa Rican, North American, and Puerto Rican children with asthma. In the most recent of these studies, [174] vitamin D insufficiency or deficiency (a serum 25[OH] D concentration <30 ng/mL) was associated with increased odds of one or more severe asthma exacerbations in the previous year even in non-atopic children. This suggests that vitamin D affects the risk of severe asthma exacerbations through mechanisms other than regulation of allergic immune responses. Reduced vitamin D concentrations are also associated with increased airway smooth muscle mass, decreased lung function, and worse disease control in children with severe, therapy-resistant asthma [178]. A Cochrane database systematic review was undertaken on vitamin D and asthma. Meta-analysis of a modest number of trials in people with predominantly mild to moderate asthma suggests that vitamin D is likely to reduce both the risk of severe asthma exacerbation and healthcare use [179].

The latest dose –response meta-analysis of 17 prospective cohort studies found the highest circulating 25-hydroxyvitamin D was associated with decreased lung cancer risk [180]. Another meta-analysis of 22 studies found that high vitamin D (or calcium) intake and serum 25(OH)D levels correlate with lower lung cancer risk and better prognosis [181].

#### Curcumin

The phytochemical curcumin, from turmeric, has been found to be a potent anti-inflammatory agent, and has been studied in regards to its anti-tumour, antifungal and antioxidant properties [182]. Animal models have demonstrated that curcumin is a potent anti-inflammatory agent in the lungs [183] and that it may also protect against pulmonary fibrosis [184]. A number of studies have suggested that curcumin may have some protective role against the DNA damage caused by arsenic [185, 186]. In a pre-clinical renal cancer study, addition of curcumin to cancer cells exhibited a strong potential for protection against diesel exhaust and cisplatin-induced cytotoxicity [187]. Preclinical trials have also shown that curcumin inhibited POP associated cellular and DNA damage [188, 189]. It has also reversed nicotine induced liver toxicity in an animal study [190].

Pre-clinical studies have shown that curcumin can prevent cadmium-induced IL-6 and IL-8 inflammatory secretion by human airway epithelial cells. Cadmium (Cd) is a toxic metal present in the environment and its inhalation can lead to pulmonary disease including lung cancer and COPD. Curcumin could therefore potentially be used to prevent airway inflammation due to cadmium inhalation [191]. An animal model investigated the effect of Cadmium (CdCl_2_)-polluted drinking water (40 mg CdCl_2_/L) on the level of tumour necrosis factor- alpha (TNF-α) and IL-6 and found a preventative action of curcumin against Cd toxicity [192].

Specifically in COPD, curcumin has been shown in animal models to have a beneficial effect in smooth muscle cells and improve the mean pulmonary artery pressure and right ventricular myocardial infarction (RVMI) through stimulating the suppressor of cytokine signalling (SOCS) -3/JAK2/STAT signalling pathways [193]. In another model, curcumin was shown to suppress chemokines and affect corticosteroid sensitivity in COPD through modulating Histone deacetylase 2 (HDAC2) expression and its effect on histone modification [194]. Another animal model showed that curcumin attenuates alveolar epithelial injury in COPD, which may be partially due to the down-regulation of Protein 66 homologous- collagen homologue (p66Shc) [195]. In a randomised, double blinded, parallel group study in patients with mild COPD and raised LDL cholesterol, 90mg curcumin was found to reduce the α1-antitrypsin–low-density lipoprotein (AT-LDL) complex, thus reducing risk of future cardiovascular events [196].

In other patients, a population based study of 2478 people found that people taking dietary curcumin through eating curry had better pulmonary function. The mean adjusted FEV_1_ associated with curry intake was 9.2% higher among current smokers, 10.3% higher among past smokers, and 1.5% higher among non-smokers [197]. In 89 patients who had poor pulmonary function due to sulphur mustard, curcumin (1500 mg/day) + piperine (15 mg/day) or a placebo were given for 4 weeks. The active supplement reduced systemic oxidative stress and clinical symptoms, and also improved health related quality of life [198].

The anti-cancer effects of curcumin against lung cancer have been investigated in vitro and in vivo xenograft mouse models. The key pathways appear to be downregulation of NF-ĸB, modulation of miRNA pathways with inhibition of caspase-3 as well as inhibition of PI3K/AKT pathways. In addition curcumin can act both as a chemo- and radio-sensitizing agent in lung cancer [199].

#### N-acetylcysteine

N-acetylcysteine (NAC) supplementation has been shown to attenuate airway responsiveness by 42% in individuals with airway hyper-responsiveness following inhalation of diesel exhaust compared with filtered air [200] and a pre-clinical study demonstrated N-acetyl-L-cysteine supplementation in smoke exposed rats showed antioxidant protective effects [201]. A meta-analysis and systematic review found long-term NAC therapy may reduce risk of patients COPD exacerbation [202]. An ex-vivo study found that NAC reduced COPD exacerbation induced by lipopolysaccharide (LPS) [203]. Supplementation of NAC, 600mg once a day and a 20 minute daily walk in addition to regular treatment improved quality of life in stable COPD patients [204]. However in another trial, treatment with NAC elevated plasma glutathione but did not modulate central or peripheral components of the O_2_ transport pathway and did not therefore improve exercise tolerance in patients with mild COPD [205]. There is little evidence of any benefits such as reducing lung cancer risk.

#### Fats

Omega-3 oils (or n-3 polyunsaturated fats-PUFAs) have received much attention due to their ability to reduce inflammation, and for its anti-coagulant properties, thus reducing risk of cardiovascular diseases. Two randomised controlled studies have investigated early life fish oil dietary supplementation in relation to asthma outcomes in children at high risk of atopic disease (at least one parent or sibling had atopy with or without asthma). In a study, powered only to detect differences in cord blood, maternal dietary fish oil supplementation during pregnancy was associated with reduced cytokine release from allergen stimulated cord blood mononuclear cells. However, effects on clinical outcomes at one year, in relation to atopic eczema, wheeze and cough, were marginal [206] In a second study, fish oil supplementation started in early infancy with or without additional house dust mite avoidance, was associated with a significant reduction in wheeze at 18 months of age. By five years of age fish oil supplementation was not associated with effects on asthma or other atopic diseases [207]. In the absence of any evidence of benefit from the use of fish oil supplementation in pregnancy, the British Thoracic Society SIGN 2016 guidelines do not recommend it as a strategy for primary prevention of childhood asthma [208].

There are recent studies which use omega-3 oils to combat the effects of pollution. Animal models of fine particle matter pollution, demonstrated that omega-3 oils prevented and improve inflammation caused by these fine particles [209] with a further pre-clinical study showing that omega-3 oils reduced the oxidative damage in the intestines after heavy metals ingestion [210].

For the early and milder forms of allergic asthma, dietary supplementation with long-chain polyunsaturated fatty acids (LCPUFA), predominantly fish oil-associated eicosapentaenoic (C20:5 ω-3) and docosahexaenoic acid (C22:6 ω-3), and distinct crop oil-derived fatty acids have been proposed to provide a sustainable treatment strategy [211, 212]. C20:5 and C22:6 ω-3 fatty acids inhibit cyclooxygenase (COX) activity and decrease eicosanoid synthesis from amino acids. [213]. They also suppress immunoglobulin (Ig) E production and thereby reduce airway inflammation and bronchoconstriction in asthma [214]. In 2002 a Cochrane database review concluded that there was insufficient evidence to recommend fish oil supplementation for the treatment of asthma [215].

In addition to immune-controlling prostaglandins, leukotrienes, and thromboxanes, specialised mediators, such as lipoxins, resolvins, protectins, and maresins are metabolised from different LCPUFA, which actively resolve inflammation. Where people with asthma are allergic to pollutants and other allergens, omega-3 and some omega 6 oils also act to reduce inflammation, rebuilding fatty acid homeostasis in cellular membranes, modifying eicosanoid metabolic pathways, thus reducing clinical symptoms [216, 217]. Most recently, an animal study compared the effects of olive oil, coconut oil and fish oil. Although fish oil protected against O3 induced vascular damage, it increased pulmonary injury/inflammation and impaired lipid transport mechanisms [218].

A Mediterranean diet has long been suggested as the most ‘healthy’ diet to follow and its health benefits are largely attributed to the content of fibre, antioxidants, protein, and moderate amounts of fat-predominantly from mono-unsaturated (MUFA) and omega-3 PUFA. Airway inflammation during asthma may be modulated by dietary intake [144, 219–221]. Fruit, vegetables and their antioxidants may lower airway inflammation [144, 220]. Fruit and vegetable intake was inversely associated with IL-8 protein in nasal lavage of asthmatic children [220]. The Mediterranean diet does offer some protection against the effects of tobacco smoke in smokers and passive smokers [222].

In contrast, high fat intake comprising of a low intake of n-3PUFAs with a corresponding increase in intake of n-6PUFAs is characteristic of the Western diet, can cause an increase in airway inflammation. This change has been linked to increasing rates of allergic disease and asthma [151]. Consumption of a high-fat mixed meal has been shown to increase sputum neutrophils 4 h post-meal in patients with asthma [223], as well as activation of a number of genes in sputum involved in “immune system processes”, such as TLR4, indicating an increase in airway inflammation [221]. Reduction of dietary saturated fat intake was associated with a reduction in neutrophilic airway inflammation in asthmatics [222]. In adults with severe asthma, higher fat and lower fibre intakes have been associated with increased eosinophilic airway inflammation [219].

In a COPD randomised placebo controlled trial of 86 patients, an omega-3, vitamin D and leucine supplement drink was given to half the group for 4 months alongside high intensity training, whilst the other half undertook the exercise alone. The population had moderate airflow limitation, low diffusion capacity, normal protein intake, low plasma vitamin D, and docosahexaenoic acid. There were significant differences after 4 months favouring the supplement group for body mass, plasma vitamin D, eicosapentaenoic acid, docosahexaenoic acid and number of steps [224].In a meta-analysis of 8 prospective cohort studies looking at PUFA and lung cancer risk, the team concluded that PUFA intake had little or no effect on lung cancer risk. PUFA intake might play a small role in lung cancer prevention in women, but this is unclear [225].

#### Choline

Choline is a lipotropic agent involved in several biological functions (eg, neurotransmitter production, signalling lipids, and components of structural membranes), and as a methyl group donor [226]. Dietary sources of choline include meat, liver, eggs, poultry, fish and shellfish, peanuts, and cauliflower. Choline deficiency is associated with neurological disorders, cardiovascular diseases, and inflammation [227].

Intranasal or oral administration of choline has been shown to reduce the number of eosinophils and reactive oxidant species in bronchoalveolar lavage fluid in a murine model of allergic airway disease [228]. In human studies, 76 asthma patients were recruited and treated with a choline supplement (1500 mg twice) or standard pharmacotherapy for 6 months in two groups. The patients were evaluated by clinical, immunologic and biochemical parameters. The treatment with choline showed significant reduction in symptom/drug score and improvement in FEV_1_ compared to baseline or standard pharmacotherapy. Choline therapy significantly reduced IL-4, IL-5 and TNF-alpha level as compared to baseline or standard pharmacotherapy after 6 months (*p*<0.01). Blood eosinophil count and total IgE levels were reduced in both of the treatment groups. [229]. In a cross-sectional survey that enrolled 1514 men and 1528 women with no history of cardiovascular disease (the ATTICA Study), fasting blood samples were collected and inflammatory markers were measured. Compared with the lowest tertile of choline intake (<250 mg/d), participants who consumed >310 mg/d had, on average, 22% lower concentrations of C-reactive protein (*P* < 0.05), 26% lower concentrations of IL-6 (*P* < 0.05), and 6% lower concentrations of tumour necrosis factor-alpha (*P* < 0.01). These findings were independent of various sociodemographic, lifestyle, and clinical characteristics of the participants [230]. This suggests that choline might attenuate allergic inflammation in general and airway inflammation in particular.

#### Dietary recommendations

Dietary recommendations from the British Thoracic Society and the Scottish Intercollegiate Guidelines Network, 2016 [208]:

- Weight reduction is recommended in obese patients to promote general health and to reduce subsequent respiratory symptoms consistent with asthma. (Grade C).
- Obese and overweight children should be offered weight-loss programmes to reduce the likelihood of respiratory symptoms suggestive of asthma (Grade C).
- Weight-loss interventions (including dietary and exercise-based programmes) can be considered for overweight and obese adults and children with asthma to improve asthma control (Grade B).

Unfortunately, despite the evidence we present there are no official recommendations for using diet or supplements to help prevent COPD and lung cancer.

## Conclusion

There is increasing evidence to suggest that carotenoids, vitamin D and vitamin E help protect against pollution damage which can trigger asthma, COPD and lung cancer initiation. Vitamin C, curcumin, choline and omega-3 fatty acids may also have a protective role. The Mediterranean diet appears to be of benefit to the airways, but there is no evidence of benefit in protecting against air pollution, except for tobacco smoke. Undoubtedly robust randomised studies are required however it is very difficult to design such studies due to the confounding factors of diet, obesity, co-morbid illness, medication and environmental exposure. Whilst such studies are being designed it would seem appropriate to consider making dietary recommendations and consider the role of appropriate supplementation in predisposed/at-risk individuals. Novel approaches to the mitigation of global chronic respiratory disease burden are urgently required.

## Abbreviations

AhR: Aryl hydrocarbon receptors
AT-LDL: α1-antitrypsin–low-density lipoprotein
C20:5 ω-3: Eicosapentaenoic
C22:6 ω-3: Docosahexaenoic acid
Cd: Cadmium
COPD: Chronic obstructive pulmonary disease
COX: Cyclooxygenase
FEV_1_: Forced expiratory volume
FVC: Forced vital capacity
HDAC2: Histone deacetylase 2
Ig: Immunoglobulin
IL: Interleukin
LCPUFA: Long-chain polyunsaturated fatty acids
LPS: Lipopolysaccharide
MUFA: Monounsaturated fatty acids
NAC: N-acetylcysteine
NO_2_: Nitrogen dioxide
O3: Ground level ozone
p66Shc: Protein 66 Src homologous- collagen homologue
PAHs: Polycyclic aromatic hydrocarbons
PBDEs: Polybrominateddiphenyl ethers
PBMC: Peripheral blood mononuclear cell
PCBs: Polychlorinated biphenyls
PM: Particulate matter
POPs: Persistent organic pollutants
RCT: Randomised controlled trial
ROS: Reactive oxygen species
RVMI: Right ventricular myocardial infarction
SO_2_: Sulphur dioxide
SOCS: Suppressor of cytokine signalling
Th: T-helper
TNF-α: Tumour necrosis factor- alpha
UFP: Ultrafine particles
WHO: World Health Organisation
